# Anti‐inflammatory effects of bay laurel (*Laurus nobilis* L.) towards the gut microbiome in dextran sodium sulfate induced colitis animal models

**DOI:** 10.1002/fsn3.3946

**Published:** 2024-01-08

**Authors:** Nazeha A. Khalil, Nora A. ALFaris, Jozaa Z. ALTamimi, Isam A. Mohamed Ahmed

**Affiliations:** ^1^ Nutrition and Food Sciences Department, Faculty of Home Economics Menoufia University Menoufia Egypt; ^2^ Department of Physical Sports Sciences, College of Sports Sciences & Physical Activity Princess Nourah bint Abdulrahman University Riyadh Saudi Arabia; ^3^ Department of Food Science and Nutrition, College of Food and Agricultural Sciences King Saud University Riyadh Saudi Arabia; ^4^ Department of Food Science and Technology, Faculty of Agriculture University of Khartoum Shambat Sudan

**Keywords:** colonic fermentation, colonic microbiota, functional food, inflammatory bowel diseases, probiotics

## Abstract

Bay laurel (*Laurus nobilis* L.) contains active antioxidative phenolic components that are beneficial to human health. However, none was examined and reported utilizing health effects related to inflammatory bowel diseases (IBD) mainly ulcerative colitis (UC) in correlation to gut microbiota (GM). Thus, the current study aimed to investigate the impacts of bay leaves on UC albino rats targeting on the GM composition and their metabolites production (i.e., short‐chain fatty acids; SCFAs) for improving the gut barrier functions. UC models were induced by supplementing 5% DSS into their drinking water. The models were then divided randomly for the diet with 1%, 2%, and 3% of bay leaves, as well as two control studies (positive and negative). Colon‐to‐body weight ratio was used as an indicator for the presence of edema tissue. From the collected fecal samples at 0, 24 h, and final day, the population changes of gut microbiota (*Lactobacillus*, *Bifidobacteria*, *Clostridium*, and sulfate‐reducing bacteria) and SCFAs production were evaluated using fluorescence in situ hybridization (FISH) and gas–liquid chromatography (GC). The colon‐to‐body weight ratio of the rat models consuming 2% and 3% bay leaves was found to be significantly lower with better recovery of colonic function. Models consuming 3% bay leaves showed the best treatment effects on GM compositions; promoting the growth of *Bifidobacteria* and *Lactobacillus* in addition to producing high butyric acid levels*.* Meanwhile, the number of *Clostridium* and SRB was significantly reduced. Conclusively, consuming bay leaves brought significant colon health benefits other than stimulating appetite for a better taste.

## Graphical abstract

Bay laurel (*Laurus nobilis* L.) contains active antioxidative phenolic components that are beneficial to human health. Consuming bay leaves brought significant colon health benefits other than stimulating appetite for a better taste.

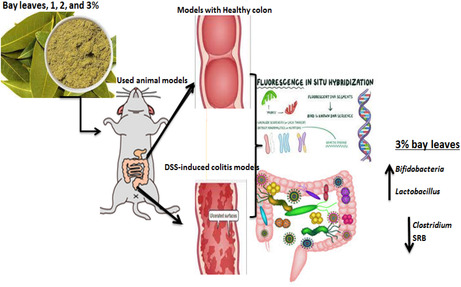

## 1 INTRODUCTION

Bay laurel (*Laurus nobilis* L.) leaves are the most important herbal spices that have been used for several years to enhance the flavor of food in addition to their nutritional and health curing properties (Sharma et al., 2012). They have been used in traditional medicine to improve nervous system functions, regulate body metabolism, and prevent blood/glucose‐related conditions like diabetes and cardiac diseases (Dobroslavić et al., 2022; Mohammed et al., 2020). They also protect the body by fighting infection, preventing cancer, strengthening bones, reducing stress, improving digestion, protecting against diseases, curing colds, protecting oral health, and balancing hormones (Casamassima et al., 2017; Dobroslavić et al., 2022). Bay leaves also exhibited anti‐oxidative, anti‐microbial, and anti‐inflammatory properties, anti‐atherosclerotic, anti‐cancer, and enhancing the central nervous system (Mandal et al., 2023). In addition, bay leaves are rich in nutrients mainly, potassium, iron, vitamin A, vitamin C, carbohydrates, calcium, dietary fibers, sodium, fats, proteins, chlorogenic acid, catechins, procyanidins, and quercetin glycosides (Batool et al., 2020; Mandal et al., 2023). Regarding such different nutrient compositions in association with colon health; previously published data explained that most of such chemical compositions, mainly, calcium and magnesium are required for gut well‐being and different protective effects as they can help for the maintenance of epithelial cell tight junctions (Bielik & Kolisek, 2021; Claverie‐Martin, 2015; Yang et al., 2020). In addition, magnesium/calcium act as the molecular basis for inflammatory response leading to the modulation of intracellular calcium levels and oxidative stress (Mandal et al., 2023). Therefore, consuming rich calcium and magnesium sources such as bay leaves could help and be in advance for achieving gut health. However, the quantity of bay leaves required to be consumed to obtain such health benefits has yet to be reported.

Incorrect consumption of herb and spices could alter the gastrointestinal microbiota, which might negatively affects human colonic health (Seralini, 2023). For instance, the occurrence of inflammatory bowel diseases (IBD) namely ulcerative colitis (UC) has been linked to an alteration in the intestinal bacteria compositions and metabolite production (Khalil et al., 2014). Many clinical and experimental researchers claimed that dysbiosis plays a vital role in the etiologic and pathogenesis of different diseases such as T2D (type 2 diabetics) and IBD, especially in patients with UC conditions (Khalil et al., 2021). Another important factor that leads to UC is the diets metabolized by the colonic microbiota and the metabolic products such as short‐chain fatty acids (SCFAs) (Qin et al., 2022). The SCFA resulting from the dietary fermentation by colonic microbiota, therefore, contributes to the anti‐inflammatory effects, which induce the immune response regulators within the intestinal mucosa cell surface (Liu et al., 2022). Additionally, SCFAs bring many beneficial health benefits such as decreasing the intestinal pH, providing energy for colonocytes, stimulating the blood flows, trans‐epithelial chloride secretion, and stimulating the colonic epithelial cell proliferation, which then prevents IBD (Seralini, 2023).

In UC patients, one of the most commonly found colonic microbiota is the sulfate‐reducing bacteria (SRB). The SRB causes damage to gut mucosa, which inhibits cytochrome oxidization by producing hydrogen sulfide (H_2_S) (Khalil et al., 2014; Kushkevych et al., 2019). A higher concentration of H_2_S was found in the fecal matter of UC patients who have 50% more SRB such as *Clostridium* spp. than normal people (Khalil et al., 2014). Accumulation of the pathogenic *Clostridium* causes damage in intestinal mucus, which the associated with gastrointestinal disorders and diseases such as colon cancer. This negative condition of the colonic microbiome can be resolved by the modulation of beneficial bacteria known as probiotics. One of the most commonly known probiotic strains is *Bifidobacteria* and *Lactobacillus*; however, those strains are un‐attempted in UC patients (Chen et al., 2021). The presence of beneficial bacteria in the gut stimulates the strength of gut barrier function, which reduces inflammation (Illescas et al., 2021). Thus, the alteration of gut microbiota plays an important role in the etiology of UC. This can also be achieved by manipulation and modulation of diets as a therapeutic option in addition to the required cross talk between the epithelial cells and microbiota.

To date, many different studies have been done globally to improve the understanding of IBD pathogenesis in association with dietary health benefits (Quaglio et al., 2021). Bay leaf extract or powder consumption was found to reduce cholesterol levels, glycemic index, blood lipid profile (glucose, total cholesterol, LDL cholesterol, triglycerides), and liver enzymes (ALT and AST) and improved the health conditions of different models (Casamassima, et al., 2017). A study with IBD model (on a murine dextran sodium sulphate; DSS) fed on bay laurel extract shown anti‐inflammatory function significantly (inhibiting nuclear factor‐κB activation) in addition to tissue morphology improvement nearly to tissues from control animals with an effective anti‐inflammatory treatments for IBD conditions (Correa & Orland, 2023). However, there is no study focused on effect of bay leaves on gut microbiota associations. In addition, no long‐term data is available regarding inflammatory models in correlation to colonic microbiome composition and activities in association with bay leaves powder consumption. Therefore, here, a long‐term study has been carried out and aimed to investigate the possible health effects of ground powdered bay leaves in different concentrations as natural supplementations to IBD animal models; exploring their uses associated with human health benefits driven by colonic microbiota manipulations.

## 2 MATERIALS AND METHODS

### 2.1 Plant materials

Dried bay leaves were purchased from the local market of Cairo, Egypt. Sample was then ground into a powder then kept in a dry cool environment until further analyses and studies.

### 2.2 Chemicals and reagents

All chemicals used were analytical grade from the Pharmaceutical Science Laboratory, National Research Centre, Giza, Egypt. Starch‐feed experimental animal models (i.e., rats) were obtained from Edwic, Egypt. Casein, cellulose, minerals, and vitamins used were purchased from Roche Vitamins and Fine Chemicals (Hague Rd, USA). Cholic acid used in this study was purchased from Sigma Aldrich (New York, USA).

### 2.3 Animal models

A total of 30 albino male rats were obtained from the Vaccine and Immunity Organization, Ministry of Health's Helwan Farm Cairo, Egyptian. The in vivo experiments were carried out at the Faculty of Home Economics, Menoufia University under the ethical approval numbered MUFHE/F/NFS/1/22. All rat models were weighed from a range of 124 to 135 g. They were housed individually in separate cages filled with hardwood chip bedding and maintained in a controlled environment with free access to water and diet to adapt to the experimental conditions (12 h light/dark cycle in a temperature‐controlled animal room at 22–25°C). The diet of the models was formulated using the standard basal control diet according to the American Institute of Nutrition's recommendation for rodent growth. All models were housed for 1 week for adaptation before introducing to any treatment. The basal diet composition used was prepared as described in our previous study (Aljutaily et al., 2022).

### 2.4 Ulcerative colitis induction

Dextran sodium sulfate (DSS; 5%) was used to induce UC conditions by adding to the drinking water of the in vivo models. DSS is toxic to the colonic epithelium, particularly to the basal crypts, which then increase the intestinal epithelium apoptosis that ends up in UC condition (Lin et al., 2019). Additionally, the DSS additions show previously epithelial damage that including ulcerations with extensive neutrophil infiltration as indicators for IBD (Correa & Orland, 2023).

Thus, the DSS‐induced colitis models were prepared as demonstrated in a previous study (Vetuschi et al., 2002). In, the structural and ultra‐structural features of DSS‐induced colitis in rats are similar to those in humans with UC conditions. Generally, rat models were administrated with 5% DSS‐induced drinking water for 1 week, except for the control healthy group (−ve) with optimal health conditions (without DSS). The rat models were then divided into groups randomly (*n* = 6) and fed with specific diets supplemented with different bay leaves concentration.

### 2.5 Experimental study design

The diet of the rat models were specified according to Table 1 in different groups with different concentration of powdered bay leaves added to the basal dietary meal after being divided into different rat groups randomly; depending on the consumed levels as in Table 1. The in vivo study was performed for up to 7 days with the specific diet plan for further analyses.

**TABLE 1:** Experimental design of rat model groups supplemented different dietary levels.

Group No.	Sample code	Basil diet	Bay leaves	Description
Group 1	G1; Control (−ve)	100%	0%	Healthy normal rats fed with basal diet without bay leaves.
Group 2	G2; Control (+ve)	100%	0%	UC rats fed basal diets without any bay leaves.
Group 3	G3	99%	1%	UC model fed with a basal diet supplemented with 1% bay leaves
Group 4	G4	98%	2%	UC model fed with a basal diet supplemented with 2% bay leaves
Group 5	G5	97%	3%	UC model fed with a basal diet supplemented with 3% bay leaves

*Note*: UC represents ulcerative colitis conditions induced by DSS.

Table 1 indicated the rat groups named and described in the current study with every used treatment. Two used control groups; negative healthy (−ve), positive UC (+ve) were used in addition to three UC groups in different bay leaves supplemented levels (1–3%).

### 2.6 Body weight gain (BWG) and colon/body weight ratio

The initial and final body weights of the rat models were measured and recorded before and after the models were provided with a specific diet plan. The body weight gain (BWG %) of the rat models was calculated as described previously (Aljutaily et al., 2022).

At the end of the experiment (7th day), all rat models were sacrificed and their colons were weighed and recorded. The colon/body weight ratio was calculated and used as an indicator for inflammatory infiltrates and the existence of edema tissue (Lin et al., 2019).

### 2.7 Colonic microbiota determination

Fresh fecal samples on three different occasions of the rat models were collected before the specific diet provided as 0‐h/Day‐0 samples. After 24 h provided with a specific diet, fecal samples were collected again for colonic microbiota enumeration. The in vivo study was carried out for a total of 7 days. The rat models were then sacrificed and the fecal samples at Day‐7 were collected as a final stage of the experiment for colonic microbiota enumeration. Bacterial enumeration was performed using the fluorescent in situ hybridization (FISH) technique as described in a previous study (Khalil et al., 2021).

The population of *Bifidobacteria*, *Lactobacillus*, *Clostridium*, and SRB were enumerated using the synthetic oligosaccharide probe with a specific region of 16 s rRNA molecule labeled with fluorescent dye Cy3 for fluorescent microscopy enumeration. Briefly, diluted fresh fecal samples (20 μL) were applied to each well of a six well PTFE/poly‐L‐lysine coated slide (Tekdon Inc., Myakka City, USA). The slides were dried in a drying chamber at 45°C for 15 min. Slides were then dehydrated using an alcohol series (50%, 80%, and 96% v/v ethanol) for 3 min in each concentration. Finally, slides were dried for 2 min before the addition of the hybridization mixture (50 μL; consisting of 5 μL probe and 45 μL hybridization buffer) that was carried out at the appropriate temperature for each probe for 4 h in a hybridization oven. Slides were washed for 15 min in the dark at the appropriate temperature for the probes being used (either 46 or 50°C). Then they were dipped in ice‐cold distilled water for 2–3 s. Finally, slides were dried then a cover slip (20 mm, thickness No 1, VWR, Lutterworth, UK) was applied for each slide then they all were stored in a dark box for fluorescent cell counting by microscopy.

### 2.8 Short‐chain fatty acids (SCFAs) evaluations

The fresh fecal samples (0‐h/Day‐0, 24 h, and Day 7 as the final stage) were analyzed for the production of short‐chain fatty acids (SCFAs). The SCFAs were evaluated using the gas–liquid chromatographic method (Khalil et al., 2014) where the supernatant samples were dispensed into Hicrom GC vials (2 mL) which were immediately sealed with rubber caps and analyzed with an HP 5890 series II GC system (Hewlett Packard, Palo Alto, California) using Helium as gas carrier with a flow rate of 2.5 mL/min. The elution times and injector flame ionization detector (FID) were maintained at 240°C and at 250°C while the column at 140°C and all data were recorded and all data were analyzed using principal component analysis and fatty acid concentrations were calculated by comparing their peak areas with those of the standards. In, acetic acids, propionic acid, and butyric acid were used as standard calibration references.

### 2.9 Statistical analysis

Statistical significance between all the experimental groups was analyzed using the SPSS package for one‐way analysis of variance (ANOVA). The mean comparison was performed using Duncan's multiple ranges as a post hoc test. The data were expressed as Mean ± SD (Standard Deviation) at *p* ≤ .05.

## 3 RESULTS

### 3.1 Body weight gained

Table 2 disclosed the initial and final body weight of all rat models provided with a specific diet plan according to Table 1 mentioned above. The differences in the body weight gain (BWG %) of all rat models were also calculated and presented in Table 2.

**TABLE 2:** Changes in body weight gain (BWG%) of rat models before and after being provided with a specific diet.

Group of the rat model	Initial body weight (g)	Final body weight (g)	Differences (final‐initial)	BWG %
G1; Control (−ve)	130.9 ± 1.75^a^	135.3 ± 1.50^a^	4.375	3.37
G2; Control (+ve)	130.1 ± 3.40^a^	124.3 ± 2.32^b^	−5.83	−4.48
G3; 1% Bay leaves	129.5 ± 3.47^a^	132.3 ± 2.90^a^	2.82	2.18
G4; 2% Bay leaves	130.1 ± 3.29^a^	132.6 ± 2.58^a^	2.58	1.98
G5; 3% Bay leaves	130.2 ± 2.22^a^	134.5 ± 2.46^a^	4.32	3.32

*Note*: Data was presented in the form of mean ± SD. Values in the same column carrying different superscript letters were significantly different (*p* ≤ .05). G1; Control (−ve): Healthy normal rats fed with basal diet without bay leaves; G2; control (+ve): UC rats fed with basal diets without any bay leaves; G3: UC model fed with a basal diet supplemented with 1% bay leaves; G4: UC model fed with a basal diet supplemented with 2% bay leaves; G5: UC model fed with a basal diet supplemented with 3% bay leaves.

From Table 2, it is found that G1 of the control animal groups with normal healthy conditions (−ve) has the highest body weight levels (135.3 g) at the end of the experiment. The body weight of G1 rat models increased by about 3.37% from the initial body weight. Similarly, rat models from G3, G4, and G5 fed with bay leaves also showed an increase in body weight. Particularly, in G5 fed with a 3% bay leaves diet, the rat models showed the highest increase in body weight at about 4.5 g. When compared to G2; the unhealthy rat models fed with a non‐bay leaves diet (+ve), both healthy rat models (G1) and UC rat models fed with bay leaves diet (G3, G4, and G5) have a significant increase in body weight (*p* ≤ .05; Table 2). Whereas, for G2 with UC animal models fed normal basal diets without any bay leaves supplementation; their body weight differences indicated a reduction at approximately 5.83 g.

### 3.2 Colon/body weight ratio

**FIGURE 1:**
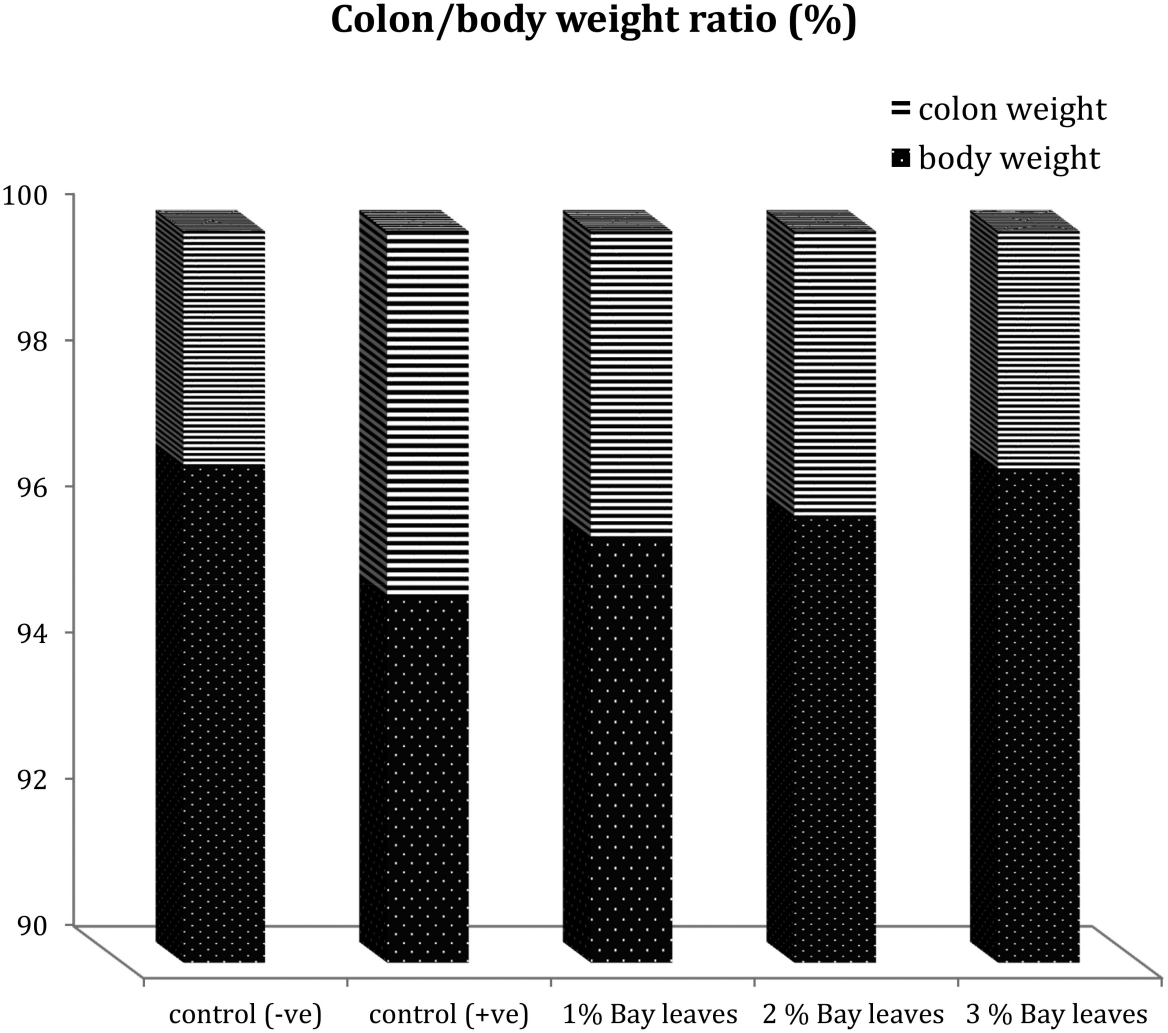
Comparison of the colon/body weight ratio between healthy and UC used animal models. Control (−ve): Healthy normal rats fed with basal diet without bay leaves; control (+ve): UC rats fed with basal diets without any bay leaves; 1% Bay leaves: UC model fed with a basal diet supplemented with 1% bay leaves; 2% Bay leaves: UC model fed with a basal diet supplemented with 2% bay leaves; 3% Bay leaves: UC model fed with a basal diet supplemented with 3% bay leaves.

Ulcerative colitis (UC) is a disease commonly accompanied by crypts lost and low levels of goblet cells. In short, these are the conditions indicating the presence of inflammatory infiltrates and edema tissue (Khalil et al., 2014). Therefore, in this study, the colon/body weight ratio was used as an indicator or index for the presence of edema tissue. The colon/body weight ratio was calculated and recorded in Figure 1.

Results in Figure 1 indicated that the UC animal models (G2; Control +ve) have the highest value of colon/body weight ratio so such models are at the highest risk for the development of edema tissue in additionally to the occurrence of epithelial cell apoptosis that may end up with alteration of the epithelial barrier. The addition of bay leaves may acts as a source of dietary fibers rather than purely enhancing the flavor of the delicacies and that may affect the colon/body weight ratio. The colon‐to‐body weight ratio is lower in G4 (fed with 2% bay leaves) and G5 (fed with 3% bay leaves) when compared to both control groups (G1 and G2). Such impacts indicate that the consumption of bay leaves has positive health effects on the colon/body weight ratio in DSS‐induced colitis. From the result, it is also observed that the colon‐to‐body weight ratio in UC models also has a dose‐independent manner. Whereby, a diet added with a higher concentration of bay leaves has a more positive impact on the colon‐to‐body weight ratio. In this case of the study, 1% < 2% < 3% bay leaves diet, yet a higher portion of bay leaves involvement should be studied for further confirmation.

### 3.3 Gut microbiome enumerations

Any alteration of the GM composition and/or activities causes a disorder named dysbiosis and such condition then affects the host's immunity and colonic intestinal integrity, thus, resulting in chronic gut inflammation (Khalil et al., 2021).

**FIGURE 2:**
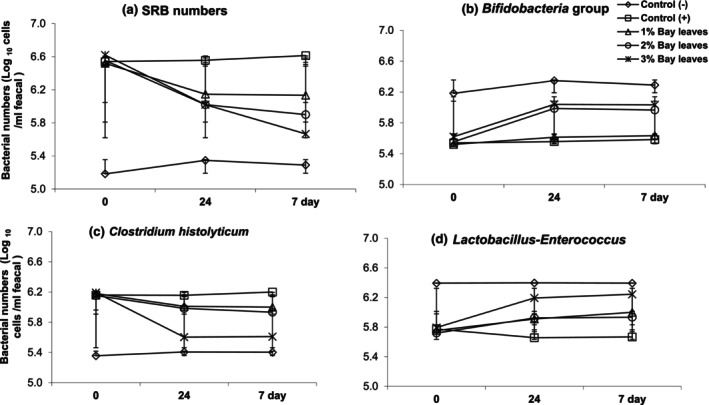
Effects of bay leaves (1%, 2%, and 3%) towards the population of colonic bacteria in vivo rat models from 0‐h, 24‐h, and 7‐days. (a) indicates the evaluated numbers of sulfate‐reducing bacteria (SRB) while (b–d) indicts the *Bifidobacteria* group, *Clostridium histolyticum* group, and *Lactobacillus‐Enterococcus* numbers, respectively. Control (−ve): Healthy normal rats fed with basal diet without bay leaves; control (+ve): UC rats fed with basal diets without any bay leaves; 1% Bay leaves: UC model fed with a basal diet supplemented with 1% bay leaves; 2% Bay leaves: UC model fed with a basal diet supplemented with 2% bay leaves; 3% Bay leaves: UC model fed with a basal diet supplemented with 3% bay leaves.

**FIGURE 3:**
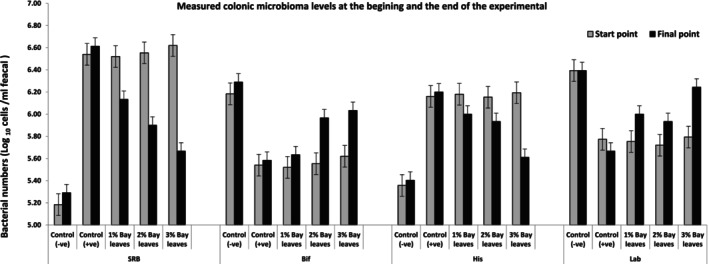
Comparison of the population of colonic bacteria in vivo rat models between the start and the end of the experiment with 7 days of treatment with a bay leaves diet (1%, 2%, and 3%). Control (−ve); Healthy normal rats fed with basal diet without bay leaves; control (+ve): UC rats fed with basal diets without any bay leaves; 1% Bay leaves: UC model fed with a basal diet supplemented with 1% bay leaves; 2% Bay leaves: UC model fed with a basal diet supplemented with 2% bay leaves; 3% Bay leaves: UC model fed with a basal diet supplemented with 3% bay leaves.

The effects of a bay leave supplemented diet on the colonic microbiota were summarized in Table 3, Figures 2 and 3. The data in Table 3 disclosed the enumeration of the targeted colonic microbiota from the treatment of the bay leave diet from 0‐h, 24 h, and Day‐7. Whereby, the targeted microbiota includes *Bifidobacteria*, *Lactobacillus*, *Clostridium*, and *SRB*. Meanwhile, Figure 2 showed the trend and population changes of the targeted colonic bacterial group.

**TABLE 3:** Effects of bay leaves consumption on colonic microbiota levels between all animal models.

Model group	Time point	Log _10_ cells/g fecal samples			
		SRB	*Clostridium*	*Bifidobacteria*	*Lactobacillus*
G1; Control (−ve)	0 h	5.18 ± 0.1^a^	5.36 ± 0.07^a^	6.18 ± 0.1^e^	6.39 ± 0.06^e^
	24 h	5.35 ± 0.34^ab^	5.41 ± 0.07^a^	6.35 ± 0.34^e^	6.40 ± 0.05^e^
	7th day	5.29 ± 0.07^a^	5.40 ± 0.14^a^	6.29 ± 0.07^e^	6.39 ± 0.07^e^
G2; Control (+ve)	0 h	6.54 ± 0.1^de^	6.16 ± 0.04^b^	5.54 ± 0.1^ab^	5.77 ± 0.03^abc^
	24 h	6.56 ± 0.09^de^	6.16 ± 0.09^b^	5.56 ± 0.09^abc^	5.66 ± 0.09^a^
	7th day	6.61 ± 0.08^e^	6.20 ± 0.12^b^	5.58 ± 0.04^abc^	5.67 ± 0.04^a^
G3; 1% bay leaves	0 h	6.52 ± 0.09^de^	6.18 ± 0.03^b^	5.52 ± 0.09^a^	5.75 ± 0.07^ab^
	24 h	6.15 ± 0.04^cde^	6.01 ± 0.03^b^	5.61 ± 0.18^abcd^	5.91 ± 0.08^cb^
	7th day	6.13 ± 0.43^cde^	6.00 ± 0.08^b^	5.63 ± 0.11^abcd^	6.00 ± 0.08^cd^
G4; 2% bay leaves	0 h	6.55 ± 0.25^de^	6.15 ± 0.04^b^	5.55 ± 0.26^abc^	5.72 ± 0.03^ab^
	24 h	6.02 ± 0.1^cd^	5.98 ± 0.05^b^	5.99 ± 0.05^cde^	5.92 ± 0.01^cb^
	7th day	5.90 ± 0.08^bc^	5.93 ± 0.1^b^	5.97 ± 0.15^bde^	5.93 ± 0.1^cb^
G5; 3% bay leaves	0 h	6.62 ± 0.15^e^	6.19 ± 0.03^b^	5.62 ± 0.15^abcd^	5.79 ± 0.03^abc^
	24 h	6.02 ± 0.1^cd^	5.60 ± 0.23^a^	6.04 ± 0.07^de^	6.19 ± 0.17^de^
	7th day	5.67 ± 0.33^abc^	5.61 ± 0.12^a^	6.03 ± 0.1^de^	6.24 ± 0.14^e^

*Note*: Data is reported in Mean ± SD; results in the same column carrying different superscript letters were different significantly (*p* ≤ .05). G1; Control (−ve): Healthy normal rats fed with basal diet without bay leaves; G2; control (+ve): UC rats fed with basal diets without any bay leaves; G3: UC model fed with a basal diet supplemented with 1% bay leaves; G4: UC model fed with a basal diet supplemented with 2% bay leaves; G5: UC model fed with a basal diet supplemented with 3% bay leaves.

From Table 3 and Figure 2, it is observed that the population of the *Clostridium histolyticum* group decreased with supplementation of the bay leaf diet. In detail, the *C. histolyticum* group of UC models decreased by 0.18 with a 1% bay leaves diet; 0.22 with a 2% bay leaves diet; and 0.58 with a 3% bay leaves diet. There are also changes in the *C. histolyticum* group of control models (i.e., G1 and G2). Whereby, the population increased by 0.04 in G1 (healthy models) and G2 (UC models with non‐BL diet).

Figure 3 demonstrates the population of SRB at the beginning and the end of our experiment. The population of SRB also showed a significant impact from the addition of bay leaves to the standard diet. Whereby, their numbers have declined by −0.39 (from 6.52 to 6.13), −0.65 (from 6.55 to 5.90), and − 0.95 (from 6.62 to 5.67) Log_10_ cells/g in bay leaves supplemented diet of 1%, 2%, and 3%, respectively. The findings in this study revealed that the population of SRB declined with bay leaf additions.

Regarding the measured population of *Bifidobacteria* in Figure 2 and Table 3 at the beginning of the experiment, the in all UC animal groups was about an average of ±5.60 Log _10_ cells/g fecal samples. However, the healthy models in G1 showed a higher number of *Bifidobacteria* at about 6.2 Log _10_ cells/g fecal samples. Surprisingly, animal models provided with bay leaves supplemented diet (G3, G4, and G5) have promoted the growth of the *Bifidobacteria* group. In detail, the *Bifidobacteria* population increased by about 0.11, 0.42, and 0.41 Log _10_ cells/g fecal samples in the models provided with bay leaves diet supplemented with 1%, 2%, and 3%, respectively. However, the growth of the *Bifidobacteria* population in a 1% supplemented bay leaves is the least among all the bay leaf diets. Whereas, the animal models provided with 2% and 3% bay leaves diet has a higher growth in the population of *Bifidobacteria*.

Additionally, the *Lactobacillus* growth has been noticed by about 0.25, 0.21, and 0.55 Log _10_ cells/g fecal samples corresponding to 1%, 2%, and 3% bay leaves diet. Among the treatment diet models fed with a 3% supplemented bay leaves diet has the highest treatment effect. Meanwhile, the population of *Lactobacillus* in G1 healthy group models remained constant throughout the treatment period about 6.39 Log _10_ cells/g fecal samples. Conclusively from Table 3, Figures 2 and 3, the bay leaves supplemented diets promote the growth of probiotics in the colon of the animal models. At the same time, the bay leaves diet also lowered the population of SRB and *Clostridium* population. Among the bay leaves supplemented diet, high dosage (i.e., 3% bay leaves diet) has a better treatment effect to stimulate probiotic growth, at the same time suppress the colonic pathogen in UC models (G5) which, the number of colonic bacterial strains is similar to those in the healthy control group (G1). Therefore, it is recommended that a higher dosage of bay leaves supplementation help to reduce intestinal inflammation by increasing probiotic growth.

In conclusion, the current study also disclosed a significant increase in the population of *Lactobacillus* strains in animal models fed with the bay leaves diet (Figure 2). Thus increasing the probiotic strains of *Lactobacillus* and *Bifidobacteria* could be preventing the occurrence of colonic inflammation and gastrointestinal disorders.

### 3.4 Short‐chain fatty acids evaluations

Colonic fermentation by the gut microbiota (GM) produces beneficial metabolites known as SCFAs (Rawi et al., 2020). A healthy and balanced colonic ecosystem produces beneficial SCFAs, which then prevent IBD and other, related gastrointestinal diseases. In this study, measuring the production of SCFAs in healthy and UC models thus helps to evaluate the efficiency of bay leaves as a fermentable substrate and potential prebiotic ingredient that promotes colonic health.

**FIGURE 4:**
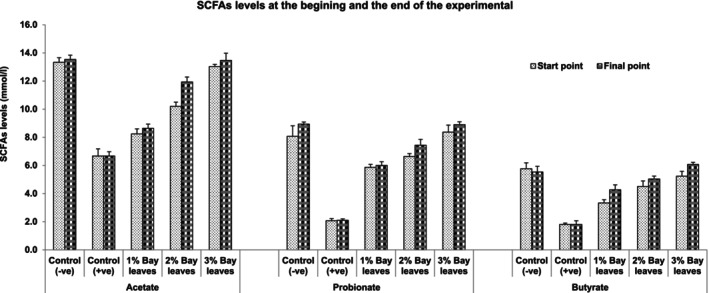
Short‐chain fatty acids (SCFAs) production from the colonic bacteria in vivo rat models between the start and the end of the experiment with 7 days of treatment with a bay leaves diet (1%, 2%, and 3%). Control (−ve): Healthy normal rats fed with basal diet without bay leaves; control (+ve): UC rats fed with basal diets without any bay leaves; 1% Bay leaves: UC model fed with a basal diet supplemented with 1% bay leaves; 2% Bay leaves: UC model fed with a basal diet supplemented with 2% bay leaves; 3% Bay leaves: UC model fed with a basal diet supplemented with 3% bay leaves.

Figure 4 showed the production of SCFAs from the animal models supplemented with bay leaf diet. Which, it is observed that acetate has the highest production, followed by propionate and then butyrate. Among the animal models, the healthy control group (G1) has the highest production of SCFAs. In contrast, the UC model provided with a diet without BL has the least production of beneficial SCFAs. From Figure 4, it is also observed that the production of acetate, propionate, and butyrate increase with the dosage of bay leaves in the diet of the models. In other words, the formation of SCFAs has a dosage‐dependent manner (3% > 2% > 1%). In this study, it is concluded that a 3% bay leaves supplemented diet is the best treatment for UC conditions. However, clinical trials and more research are to be done to mimic the human large intestine conditions for more accurate results and conclusions.

## 4 DISCUSSION

Animal body weight gain has a significant increase (*p* ≤ .05) in both healthy rats and rats who consumed bay leaves compared to the unhealthy group (induced colitis; Table 2). Indeed, similar findings were reported in a previous study (Britto et al., 2019). In, it is reported that UC rat models developed from a DSS‐induced diet have a reduction in body weight. The study proposed that this is related to the etiology and pathogenesis of the inflammation phenomenon that occurred in the gut due to exposure to DSS. The study also indicated that body weight loss is a common condition that occurred for the DSS‐induced colitis models and it is a reliable positive parameter correlated with the DSS inflammation. This is in agreement with a similar conclusion in another research comparing the body weight of the *f* DSS‐induced group and the control healthy group (Arda‐Pirincci & Aykol‐Celik, 2020). Therefore, for the significant increase in body weight of rat models in G3, G4, and G5 with fed with bay leaves diet, is likely related to the beneficial health effects of the bay leaves for its micronutrients and nutritional functionalities. Conclusively, bay leaves have positively affected the host's health regardless of the UC gut condition.

The UC animal models (G2; C + ve) have the highest value of colon/body weight ratio. This indicates that such models are at the highest risk for the development of edema tissue, as well as the occurrence of epithelial cell apoptosis that may end up with alteration of the epithelial barrier. Similar findings were reported in the study of UC patients. These patients have a high occurrence of colonic apoptosis in oxidative stress conditions. Which, this is triggered by the expression of several genes responsible for the cellular death caused by the alteration within the colonic functionalities (Zhou et al., 2017). The UC model has permitted an increase in apoptosis that leads to a breakdown of epithelial barrier function. Whereby, it facilitates the mucosal invasion of the intra‐luminal microorganisms and/or antigens. Thus, causing an abnormal and persistent epithelial hyper‐proliferation related to the development of colorectal cancers in the setting of chronic colonic inflammation (Vetuschi et al., 2002). Another study also disclosed that a high‐concentrate grain diet would induce colonic epithelial barrier disruption. This is associated with the activation of cell apoptosis in lactating goats (Tao et al., 2014).

The Mediterranean diet (MD) commonly involved abundant amounts of beneficial plant food. Thus, the gut environment of people consuming MD is usually highly associated with beneficial bacteria groups. This then improves the gut microbiota (GM) and reduces the occurrence of gastrointestinal issues among IBD patients. This can be explained by the enrichment of the beneficial bacteria; which supported the gut barrier function and eventually reduced gut inflammation. In contrast, for people who usually consume a Western diet that mainly contains pro‐inflammatory potential food, it negatively affects the gut microbiota and leads to gastrointestinal disorders (Ratajczak et al., 2023; Rawi et al., 2020). As mentioned, the consumption of MD usually involved the addition of bay leaves, which also acts as a source of dietary fibers rather than purely enhancing the flavor of the delicacies. Such impacts indicate that the consumption of bay leaves has positive health effects on the colon/body weight ratio in DSS‐induced colitis. From the result, it is also observed that the colon‐to‐body weight ratio in UC models also has a dose‐independent manner. Whereby, a diet added with a higher concentration of bay leaves has a more positive impact on the colon‐to‐body weight ratio. It is proposed that the health benefits of bay leaves are associated with the micronutrients, especially calcium and magnesium. These minerals are in great need of epithelial cell tight junction (TJ) maintenance. This is previously explained by Claverie‐Martin (2015) that epithelial cell tight junctions' disruption may cause negative impacts such as loss of epithelial integrity, mucosal injury, and increased exposure to luminal antigens, which then lead to colonic inflammation. Additionally, the study also reported that lumen‐positive voltage forces the reabsorption of magnesium and calcium. Therefore, it is recommended that oral intake of magnesium and calcium supplements is a necessary therapeutic option for UC condition. Another study also recommended Vitamin D supplements as a co‐factor for escalating intestinal calcium absorption (Christakos et al., 2020). An old study in the last two decades also concluded that both magnesium and calcium affect the epithelial tissue and open TJ in the small and large intestines in vitro models (Mineo et al., 2004). Conclusively, the consumption of bay leaves has revealed an impact on the alteration of the gut and even improves the relative inflammatory levels. Which, this is due to the presence of valuable micronutrients (calcium and magnesium).

Recent studies mentioned that dietary intake influences the GM or colonic microbiota composition and metabolism, which then contributes to human health (Rawi et al., 2020). As mentioned earlier, the consumption of the MD has a positive impact on colonic health. Using *Clostridium*, a pathogenic colonic strain, the increase in the population may be due to low consumption of MD. Whereby, MD commonly involved the addition of bay leaves. In contrast, the beneficial probiotic strains will be enhanced and decrease the occurrence of intestinal inflammation with the MD that involved bioactive compounds such as polyphenols. Whereby, the polyphenol plays an important role in antioxidant activities, which improve colonic epithelium cells' function with GM modulation; thus, decreasing the colonic *Clostridium* while increasing the levels of *Lactobacilli* and *Bifidobacterial* (Ratajczak et al., 2023).

The population of SRB again in Figure 3 also showed a significant impact from the addition of bay leaves to the standard diet. This is in line with the previous study, whereby UC patients showed a high SRB level in contrast to the healthy (Khalil et al., 2014). The current study revealed that the population of SRB declined with bay leaf additions that is proposed and related to the rich bioactive compounds in bay leaves, which promotes antioxidant activities, therefore, beneficial to prevent inflammation (Ratajczak et al., 2023).

The *Bifidobacteria* numbers in all UC animal groups were lower than the healthy models in G1 that showed a higher number of *Bifidobacteria* additionally to animal models provided with bay leaves supplemented diet (G3, G4, and G5) that have promoted the growth of the *Bifidobacteria* group. A similar observation was found in the study of Ratajczak et al. (2023). In, the bay leaves in MD have significantly stimulated the growth of probiotic strains, that is, *Bifibacteria* and *Lactobacilli*. To support this statement and conclusion, the current study also disclosed a significant increase in the population of *Lactobacillus* strains in animal models fed again with the BL diet (Figure 2). Indeed, previous studies as well revealed a vital role in the growth of *Lactobacillus* from the high dosage of polyphenol dietary (Hamody et al., 2021). In other words, polyphenols in bay leave supplemented diet promote GM, particularly the probiotic strains of *Lactobacillus* and *Bifidobacteria*. At the same time, the bay leaves diet also lowered the population of SRB and *Clostridium* population stimulating probiotic growth; at the same time suppress the colonic pathogen in UC models (G5). Therefore, it is recommended that a higher dosage of BL supplementation help to reduce intestinal inflammation by increasing probiotic growth. A similar conclusion is in agreement with the previous study regarding MD consumption among IBD patients (Chicco et al., 2021).

The final measured parameters were observed in this study are the colonic fermentation beneficial metabolites produces known as SCFAs (Rawi et al., 2020). These SCFAs play an important role in maintaining colonic health such as lowering the pH of the colonic environment thus reducing the growth of pathogenic bacteria, anti‐inflammation, preventing the production of gas that causes bloating issues, and more. It is also reported that the production of SCFAs depends on GM composition (Sarbini & Rastall, 2011). A healthy and balanced colonic ecosystem produces beneficial SCFAs, which then prevent IBD and other, related gastrointestinal diseases. A similar observation was previously reported in the work of Khalil et al. (2014). Measured SCFAs from the animal models supplemented with a bay leaf diet were acetate in the highest levels, followed by propionate and then butyrate; their production have been increased with the dosage of bay leaves in the diet of the models to conclude that a 3% bay leaves supplemented diet is the best treatment for UC conditions. However, clinical trials and more research are to be done to mimic the human large intestine conditions for more accurate results and conclusions.

## 5 CONCLUSION

The involvement of spices in the diet has been highly regarded for various health benefits, particularly in the Mediterranean diet, which affects the lifestyle quality in IBD patients. There is a serious lack of global recognition of bay leaves main ingredients and gut microbiota interactions, especially between UC conditions. From this study, it is concluded that 3% bay leaves supplemented diet showed a significant positive health effect on the body weight and colon to the body weight. In terms of the gut microbiome, a 3% bay leaves diet in UC models increases the population of *Bifidobacteria* and *Lactobacillus*, and significant suppressor in the growth of *Clostridium* and *SRB*. Additionally, the production of health‐beneficial SCFAs production also showed a positive response, especially for butyrate. UC models fed with a 3% bay leaves diet have a positive health effect similar to those in healthy models.

## AUTHOR CONTRIBUTIONS

**Nazeha A. Khalil:** Conceptualization (equal); data curation (equal); formal analysis (equal); investigation (equal); methodology (equal); visualization (equal). **Nora A. AlFaris:** Validation (equal); visualization (equal); writing – review and editing (equal). **Jozaa Z. ALTamimi:** Investigation (equal); methodology (equal); visualization (equal). **Isam A. Mohamed Ahmed:** Resources (equal); validation (equal); writing – review and editing (equal).

## FUNDING INFORMATION

This research was funded by Princess Nourah bint Abdulrahman University Researchers Supporting Project Number PNURSP2024R257, Princess Nourah bint Abdulrahman University, Riyadh, Saudi Arabia.

## CONFLICT OF INTEREST STATEMENT

The authors declare no conflict of interest.

## ETHICS STATEMENT

All procedures including animal use were approved by the official Review Board at the Faculty of Home Economics, Menoufia University under ethical approval numbered (MUFHE/F/NFS/1/22).

## Data Availability

The data supporting the conclusions of this article is included in the manuscript.
